# Automatic Classification of Cancerous Tissue in Laserendomicroscopy Images of the Oral Cavity using Deep Learning

**DOI:** 10.1038/s41598-017-12320-8

**Published:** 2017-09-20

**Authors:** Marc Aubreville, Christian Knipfer, Nicolai Oetter, Christian Jaremenko, Erik Rodner, Joachim Denzler, Christopher Bohr, Helmut Neumann, Florian Stelzle, Andreas Maier

**Affiliations:** 1 Pattern Recognition Lab, Computer Science, Friedrich-Alexander-Universität Erlangen-Nürnberg, Erlangen, Germany; 2 Department of Oral and Maxillofacial Surgery, University Medical Center Hamburg-Eppendorf, Hamburg, Germany; 3 Erlangen Graduate School in Advanced Optical Technologies (SAOT), Friedrich-Alexander-Universität Erlangen-Nürnberg, Erlangen, Germany; 4 Department of Oral and Maxillofacial Surgery, University Hospital Erlangen, Friedrich-Alexander-Universität Erlangen-Nürnberg, Erlangen, Germany; 5 Computer Vision Group, Friedrich-Schiller-Universität Jena, Jena, Germany; 6 Department of Otorhinolaryngology, Head and Neck Surgery, University Hospital Erlangen, Friedrich-Alexander-Universität Erlangen-Nürnberg, Erlangen, Germany; 7 First Department of Internal Medicine, University Hospital Mainz, Johannes Gutenberg-Universität Mainz, Mainz, Germany

## Abstract

Oral Squamous Cell Carcinoma (OSCC) is a common type of cancer of the oral epithelium. Despite their high impact on mortality, sufficient screening methods for early diagnosis of OSCC often lack accuracy and thus OSCCs are mostly diagnosed at a late stage. Early detection and accurate outline estimation of OSCCs would lead to a better curative outcome and a reduction in recurrence rates after surgical treatment. Confocal Laser Endomicroscopy (CLE) records sub-surface micro-anatomical images for *in vivo* cell structure analysis. Recent CLE studies showed great prospects for a reliable, real-time ultrastructural imaging of OSCC *in situ*. We present and evaluate a novel automatic approach for OSCC diagnosis using deep learning technologies on CLE images. The method is compared against textural feature-based machine learning approaches that represent the current state of the art. For this work, CLE image sequences (7894 images) from patients diagnosed with OSCC were obtained from 4 specific locations in the oral cavity, including the OSCC lesion. The present approach is found to outperform the state of the art in CLE image recognition with an area under the curve (AUC) of 0.96 and a mean accuracy of 88.3% (sensitivity 86.6%, specificity 90%).

## Introduction

Squamous Cell Carcinoma is a form of cancer that originates from squamous cells of the skin or mucous membranes. In the area of the head and neck, the malignant transformation of these cells leads to a worldwide incidence of 1.3 million new cancer cases per year^1,2^. Most cases of Head and Neck Squamous Cell Carcinoma (HNSCC) are already at an advanced stage when diagnosed which significantly reduces the survival rate after curative treatment^3^. The gold standard for diagnosis of HNSCC is an invasive biopsy of the lesion, followed by a histopathological assessment^4^. In addition imaging methods such as narrow band imaging^3^ and Raman spectroscopy^4,5^ are considered as emerging tools used for non-invasive detection of malign neoplasms of the head and neck. Recently, Confocal Laser Endomicroscopy (CLE)^6^, an imaging technique that has been widely used and validated in pathological tissue diagnosis of the gastrointestinal tract^7,8^, has also been studied for its potential of reliably diagnosing HNSCC *in situ* ^9,10^.

Compared to bright light endomicroscopy, CLE does not only have the advantage of an exceptionally high magnification of up to 1000x^9^, but also provides a better depth penetration^11^, allowing for diagnosis of malignancies approximately 100 microns below the surface. For this imaging technology, a fiber bundle that is connected to a laser source in the cyan spectrum (488 nm) is applied on biological tissue in cavities of the human body. A contrast agent (fluorescein) is administered to the patient by i.v. injection prior to the examination. This agent accumulates in the intercellular gaps and emits light (at 520 nm) upon excitation by the laser light, thus enabling imaging of cell outlines. The beam path, including laser source and a pinhole are constructed in such a way that light reflected from outside the focal plane is geometrically eliminated^12,13^. As both, the detector and the laser source, are in the same focal plane, the system is called ‘confocal’^8^.

Since the grayscale images of biological cells in its compound as acquired by CLE are unlike other imaging techniques, special training for the pathologist or surgeon interpreting the images is of great importance^13^, and examiner’s experience has a distinct influence on the over-all CLE performance^14^.

This technical tool has the potential to provide additional real-time information about the suspicious lesion, supportive to the clinical examination. The non-invasive, chair-side assessment of suspicious lesions without any time delay can reduce the morbidity of the patients as well as the survival rate. Consequently, the investigated system serves as an additional tool supporting the gold standard of biopsy and the following histopathological examination.

Besides this, another important field of application of an automatic classification in CLE imaging is the surgical therapy of the malignancy in terms of computer-aided surgery. Finding an adequate resection margin of a tumor is crucial for the overall success of the curative therapy. Failure to find this margin with a subsequent recurrence of the cancer is the most common cause of death for patients with HNSCC^10^. Since CLE images are taken at a greater depth than common endoscopic images, a larger field involvement beneath the surface can be detected, which has the potential to reduce morbidity and mortality after surgery^10^.

## Automatic Classification of CLE images

The image sequences acquired using CLE imaging are very different to other forms of medical images, as they display a small horizontal layer, up to 100 *μ*m beneath the surface of the probe^15^. For the present study, images were acquired using a standalone probe-based CLE system (Cellvizio, Mauna Kea Technologies, Paris, France). The probes used for imaging were CystoFlex UHD R and ColoFlex UHD, both having a similar field of view and penetration depth. The inspected area is approximately 250 *μ*m in width and height, with a total number of 576 px. Automatic classification using CLE imaging has already been proven to show valid results in clinical studies^16–18^. However, for each anatomical location, CLE images differ as the tissue under observation also differs. André *et al*. have shown that automatic image recognition using probe-based CLE can be used successfully for detection of neoplastic tissue in the colon region^16^. Their results indicate that automatic recognition can yield similar results to the diagnosis by endoscopy experts. Kamen *et al*. have successfully applied machine learning techniques on CLE images of brain tumors^17^.

Jaremenko *et al*. have first employed automatic image recognition on CLE images of the oral cavity, using the classical pattern recognition workflow with a number of textural features (local binary pattern (LBP), gray-level coocurrence matrix (GLCM) or local histogram statistics) and subsequent machine learning techniques (random forest (RF) and support vector machine) for the classification^19^. Dittberner^20^ and Rodner^21^ have shown that also segmentation-based methods have the potential to be applied to cancer recognition in CLE images of the head and neck region. They extracted the cell borders from the image and used a distance transform with successive histogram calculation in order to use the cell size as a feature for classification. On the respective data set, they reached a mean cross-validation accuracy of 74%^20^. In both cases, the number of images used in the recognition task was rather limited, calling for validation of the techniques with a substantial increase in image material.

The present study features a rather large amount of data, enabling a different class of machine learning techniques: deep artificial neural networks (DNN). While the classical, feature-based approach incorporates prior knowledge about the classification task (by proper selection of features), deep learning techniques commonly are solely calculated on the raw input data. The number of unknown parameters is much higher for a DNN approach, compared to the classical workflow, which also calls for a much higher amount of training data.

## Convolutional Neural Nets

For image recognition, one particularly successfully used method in recent years is the application of convolutional layers in these deep networks. Convolutional filters are inspired by the pattern recognition of the visual cortex, where so-called receptive fields show activations based on distinct spatial patterns of the visual scene^22^.

Methods employing Convolutional Neural Networks (CNNs) have won all major image recognition challenges (like the ILSVRC challenge^23^) and have recently also been successfully applied in the field of medical image analysis^24–27^ and even reconstruction^28^.

## Material

For the present study, *N* = 116 video sequences from 12 patients with cancer in the oral cavity were acquired at the Department of Oral and Maxillofacial Surgery (University Hospital Erlangen). Written consent was obtained from all patients and institutional review board approval was provided prior to the study (ethics committee of the University of Erlangen-Nürnberg; reference number: 243_12 B).

**Figure 1:**
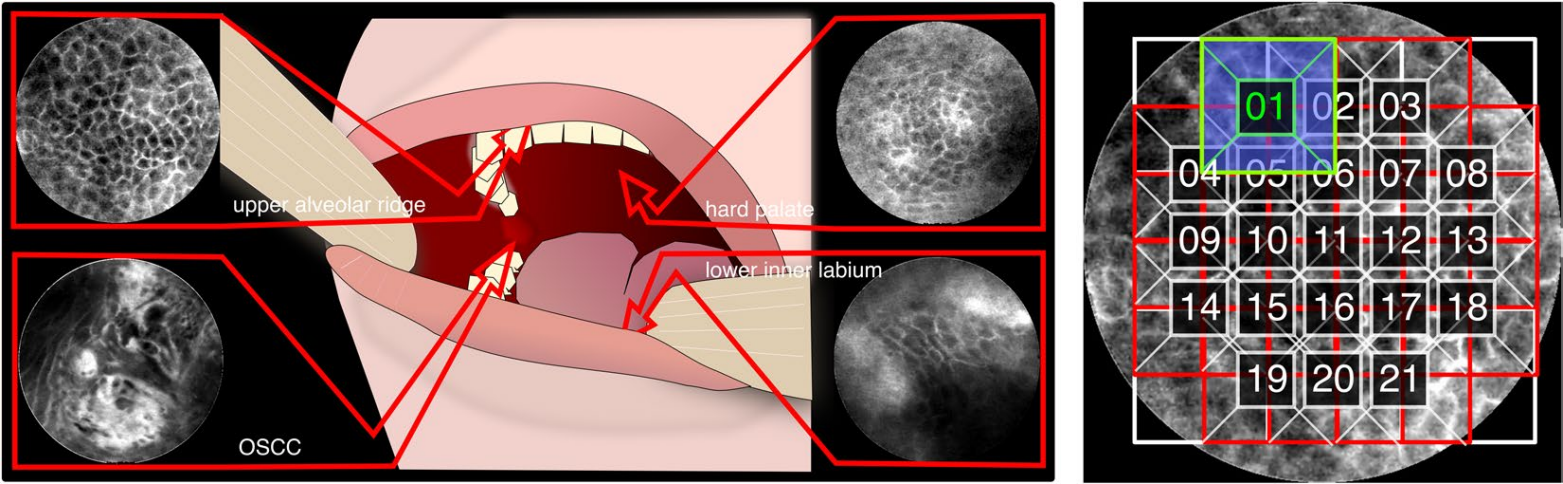
Left: CLE recording locations. Additionally, the region of the suspected HNSCC was recorded. Right: Division of (resized) image into patches of size 80 × 80 px. Only patches that were inside the image mask and had no artifact labels within them were considered for classification.

From each patient, image sequences from the suspected carcinogenic region were recorded. Additionally, images from three other (presumably physiological) regions were made: From the inner lower labium, the upper alveolar ridge and the region of the hard palate (see Fig. 1 left and Table 1). Specimen from all tumorous regions were resected after image acquisition and histologically verified by a trained pathologist. The video sequences acquired before surgery, were hand-cut by a clinician expert in order to remove parts where the instrument was not properly placed or did not show the tissue to be investigated. This resulted in approximately 11,000 images, having different image qualities and some impaired by heavy artifacts.

**Table 1:** Number of images of different regions.

Class	location	No. total	No. good images	Percentage in final data set
normal	alveolar ridge	2,133	1,951	24.71%
normal	inner labium	1,327	1,317	16.68%
normal	hard palate	955	811	10.27%
carcinogenic	various	6,530	3,815	48.33%

The most common artifact (2,659 images) in the data set was noise, ranging from slight added noise to images containing only noise. This may be related to an illumination problem, where no contrast agent is located under the probe, or the probe is not properly placed on the mucosa. Another common (1,455 images) artifact is motion artifacts, originating from movement of the probe during image acquisition, resulting in shearing, compression or elongation of the image or parts of the image. This effect severely deteriorates the image, which is why affected images were also excluded. After also excluding images with optical artifacts (such as mucus or blood drops on the probe) and images of otherwise bad quality, 7,894 images of good quality remain for the purpose of image recognition. This results in a mean image count of 658 images per patient (σ = 399). All images were either assigned to the class “clinically normal” or the class “carcinogenic”, with an almost even distribution of both classes (see Table 1).

In our opinion, the disproportionate amount of cancer images that were discarded due to noise and motion artefacts (58.4% used images in contrast to 92.3% of healthy tissue) can be attributed to the following: In the case of large tumors an uneven, rough surface is common resulting in the probe slipping off or not lying flat on the surface within the total field of vision. Furthermore, sputum/mucus and a large amount of detritus often caused by increased inflammation and cell death (e.g. ulcers) can gather there easily and build an interfering superficial layer. Also circulation conditions of cancerous or surrounding tissue can distinguish significantly from healthy conditions so that even a gently touch of the suspicious lesion can cause an immediate bleeding resulting in completely light images (because of the large amount of fluorescein) without any informative value. All these factors cause a higher rate of artifacts in cancer images in comparison to imaging of healthy tissue.

## Methods

The research was carried out in accordance with the Code of Ethics of the World Medical Association (Declaration of Helsinki) and the guidelines of the Friedrich-Alexander University Erlangen-Nuremberg.

### Patch-extraction of images

In principal, we follow the workflow of Jaremenko *et al*.^19^ in that images are divided into patches, where information is extracted and dimensionality reduced, and subsequent fusion of the information to achieve classification per image.

The images were extracted from the raw data container the CLE imaging system produces in a 16 bit grayscale format^29^. Since CLE images are often noisy, we propose to reduce processing complexity and noise in the image by scaling the image down to half the size. This way also more relevant structures are captured in a single patch.

Each CLE image has a size of 576 × 576 px. The images have a circular shape which makes processing the whole image at a time difficult. Because of this, we’re dividing the resized image (denoted **I**) into patches (denoted **P**) of size 80 × 80 px with an 50% overlap, centered around the middle of the image, resulting in 21 patches out of 1 image (see Fig. 1 right):

1 $${\bf{I}}\mathop{\longrightarrow }\limits^{patchextraction}[{{\bf{P}}}_{0},{{\bf{P}}}_{1},\ldots {{\bf{P}}}_{N{\bf{I}}}]\quad N{\bf{I}}\le 21$$

Each resulting patch *P*_*n*_ is assigned a coordinate quadruple that delimits the corners of the patch:

2 $$\overrightarrow{c}({{\bf{P}}}_{i})=[{c}_{\mathrm{1,}i},{c}_{\mathrm{2,}i},{c}_{\mathrm{3,}i},{c}_{\mathrm{4,}i}]$$

where $({c}_{1},{c}_{3})$ is the left top corner and $({c}_{2},{c}_{4})$ the bottom right corner.

Within the images with overall good image quality, a number of images have minor known, annotated artifacts (annotated as rectangles within the image), only affecting the image slightly. Patches with artifacts are removed from the image recognition task, while the rest of said images is included. This means, however, that the number of patches per image is not constant, and thus restricts the possibilities for whole-image classification.

To assume no prior knowledge about illumination of the image, all patches were whitened using a standard scaling to achieve zero mean and unity standard deviation.

### Data augmentation for training

Since CLE images have no natural orientation, a rotated CLE image is still a valid image. Because of this, we enrich the data provided to the classifier by arbitrarily, randomly rotated copies of known images. These augmented images however may not be used for testing the algorithm, since we can’t completely eliminate the possibility that the images have some inherent properties that are indeed rotation-variant, for example originating from a common hand position of the physician. We used a 2-fold augmentation, meaning that out of each original image, two randomly rotated copies were created and fed to patch extraction.

In order to avoid introducing bias for one of the classes, each classifier receives an equal distribution of both classes for training by removing augmented images of the majority class.

### Classification approaches

#### Textural feature-based classification

The approach described by Jaremenko *et al*.^19^ uses different textural features. Amongst the best scoring were features based on local binary patterns (LBP) and gray-level co-occurance matrices (GLCM), which is why these were included for comparison.

LBPs describe a pixel gray value in relationship to its neighboring pixels and were successfully used for image recognition tasks such as face recognition^30^ or cell phenotype classification^31^. Jaremenko *et al*. use rotation invariant uniform LBPs and calculate a histogram for each patch of these. Instead of using the histograms themselves as features for the classifier, the mean and standard deviation of the features over all patches of an image are used.

GLCMs, on the other side, describe the statistical occurrence of certain gray values in neighboring pixels within an image patch. From these matrices, certain features that characterize properties of the image can be calculated. Jaremenko *et al*. use the GLCM-based features described by Haralick^32^, as well as the extended features described by Baraldi *et al*.^33^.

As classification approach, support vector machine (SVM) and random forest (RF) were used.

Jaremenko *et al*. reported very convincing results, especially for GLCMs with SVM classifier (accuracy = 99. %) and also good values for LBPs (accuracy = 91.2%), on a small database of only 251 images^19^, however. GLCM-based features were calculated with different image-level configurations (8, 16 and 32 levels), and showed similar results. Since generalization of these results can’t be assumed, we included both the GLCM-features and the LBP-features in our evaluation. The approaches were evaluated on patch sizes of 80 × 80 px and 105 × 105 px, with comparable results. Vo *et al*. re-evaluated both feature sets on a much larger database of vocal cords CLE images^34^ and found comparable results for GLCM-features and LBP-features, with LBPs performing slightly better and little difference amongst the different configurations of features (i.e. image levels for GLCMs).

We included the following configurations for comparison:

1. **RF-LBP@1.0x** Random Forest-classified result using the LBP feature set (radii = [1, 3, 5], number of neighbors = [8, 16, 24], rotation invariant uniform LBPs, mean and std over all patches, number of trees = 500)
2. **RF-LBP@0.5x** equal to RF-GLCM@1.0x, but calculated on a resized (factor 0.5) image
3. **RF-GLCM@1.0x** Random Forest-classified result using the GLCM-based feature set with 16 image levels (mean and std over all patches, number of trees = 500)
4. **RF-GLCM@0.5x** equal to RF-GLCM@1.0x, but calculated on a resized (factor 0.5) image

#### Patch-based Convolutional Net Processing

Convolutional Neural Networks (CNNs) do not rely on feature extraction as a first step, but take an image as input and have feature extraction inherently within the network.

In principal, CNN machine learning can be run on the whole image as well as on patches. If patches can be considered a representative sample of the whole image, patch extraction is a beneficial approach because of the following reasons:

- Classification of patches reduces the order of the pattern recognition problem. As the number of parameters to be learned for the pattern recognition algorithm, in our case the neural network, goes quadratically with the image length and width, it is dramatically reduced. Since CNN approaches in general require a large amount of data in comparison with feature-based machine learning approaches, this is an important factor.
- Reduction of classification error. If an independent error is assumed on the result of the classification, fusion of the single patch classification results will reduce the overall error of the image classification.

In our case, we consider the whole image *cancerous* or *clinically normal*, since no sub-image labelling was performed and it was observed that the vast majority of patches show the same characteristics as the image classification.

**Figure 2:**
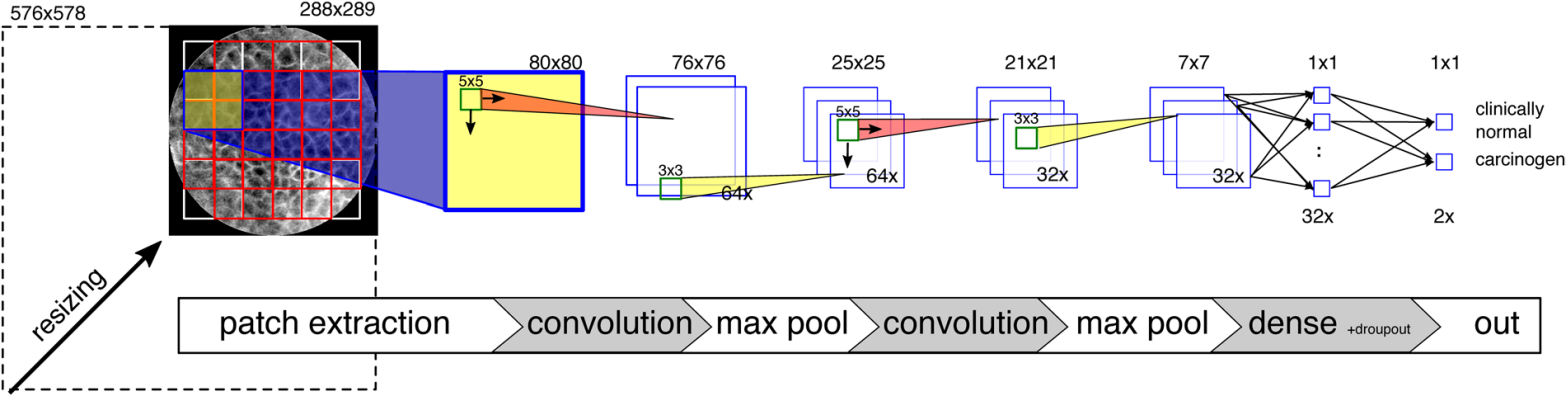
Overview of the CNN-based patch extraction and classification.

Our convolutional network is based on the LeNet-5 network proposed by Lecun^35^: A convolutional layer with 64 filters of size 5 × 5 px is followed by a max-pooling layer (3 × 3 px), another convolutional layer with 32 filters of size 5 × 5 px, another max-pooling layer (3 × 3 px), one fully connected layer with drop-out and an output layer with softmax output (see Fig. 2). The network was trained with the TensorFlow framework^36^ at an initial learning rate of 0.001 using the Adam optimizer^37^ for cross-entropy minimization. The network has 103,170 learnable parameters and was trained completely from scratch.

**Figure 3:**
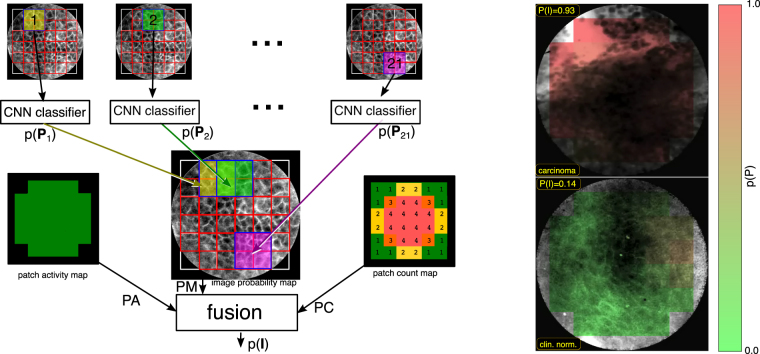
Overview of the patch probability fusion approach. Left: Overlapping patches are extracted from the image and classified. Subsequently, the image classification is fused. Right: Examples for color-coded image probability maps.

As depicted in Fig. 3 (left), the CNN-based classifier assigns each patch **P**_*i*_ an a posteriori probability $p({P}_{i})$ for the class *cancerous*. Due to symmetry of the classifier, ensured by the softmax operation at its output, this probability adds up with the probability for *clinically normal* to 100%. The extraction of overlapping patches puts a slight emphasis on those areas of the image that are covered multiple times by patches, which we however found beneficial for the overall accuracy.

This probability of a patch belonging to a cancerous image can be mapped on the image again. For this, we define first of all the area function of a patch **P**_*i*_:

3 $${A}_{x,y}({{\bf{P}}}_{{\bf{i}}})=\{\begin{array}{cc}1 & {\rm{if}}\,(x,y)\in [{c}_{1},{c}_{2}]\times [{c}_{3},{c}_{4}]\\ 0 & {\rm{else}}\end{array}$$

From this we derive the patch activity map:

4 $${{\rm{PA}}}_{x,y}=(\sum _{i}{A}_{x,y}({{\bf{P}}}_{i}))\ge 1$$

and the patch count map:

5 $${{\rm{PC}}}_{x,y}=max(\mathrm{1,}\,\sum _{i}{A}_{x,y}({{\bf{P}}}_{i}))$$

Finally, the probability map of the image is:

6 $${{\rm{PM}}}_{x,y}={{\rm{PA}}}_{x,y}\cdot {{\rm{PC}}}_{x,y}^{-1}\cdot \sum {A}_{x,y}({{\bf{P}}}_{i})\cdot p({{\bf{P}}}_{i})$$

From this we derive a scalar probability number for the image **I**:

7 $$p({\bf{I}})={(\sum _{x,y}{{\rm{PA}}}_{x,y})}^{-1}\sum _{x,y}{{\rm{PM}}}_{x,y}$$

The a posteriori probabilities of the image are thus fused into a single probability number, thus we denote the approach **patch probability fusion (ppf)** method.

#### Whole image classification using Transfer Learning with CNNs

Besides the patch-based detection of images, it is also possible to feed the complete image to the classification method. While the number of images fed to the classification training decreases, the network complexity and thus the number of free parameters increases dramatically for this approach, fueling the need for a regularization in order to prevent the network from overfitting.

**Figure 4:**
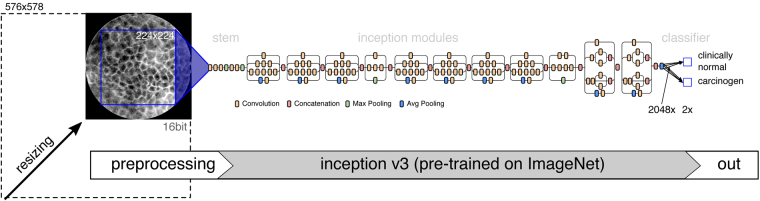
Overview of the transfer learning approach, based on Szegedy’s Inception v3^38^, pre-trained on the ImageNet database^39^.

Commonly, this problem is solved using network architectures, that were pre-trained on images of a different domain (e.g. real-world photography images or other medical images) and are then fine-tuned on a new image data set (transfer learning)^24,26^. We use the Inception v3 network from Szegedy *et al*.^38^, pre-trained using ImageNet^39^, and replace the final dense layer and softmax layer with a new two node dense layer and subsequent softmax layer (see Fig. 4).

Since CLE image data is 16 bit at a single wave length and the Inception v3 takes 8 bit RGB images, some pre-processing needs to be applied. In order to reduce 16 bit depth into 8 bit, we apply a dynamic compression: The image is scaled according to the following percentile scaling rule:

8 $${\bf{I}}{8}_{bit}=\frac{255}{{P}_{\mathrm{99.5 \% }}-{P}_{\mathrm{0.5 \% }}}\cdot (I-{P}_{\mathrm{0.5 \% }})$$

with *P*_*y*_ being the *y*th percentile of pixel intensity values within the circular view area.

The resulting 8 bit image is then mapped to a greyscale RGB image, from which the maximum square area is extracted. It is defined as a square with dimensions $w=h=\frac{2}{\sqrt{2}}\cdot {r}_{{\rm{CLE}}}$ around the center of the image, where ${r}_{{\rm{CLE}}}$ is the radius of the circular CLE view area in pixels.

In order to fit the target input dimension of 224 × 224 pixels of the Inception v3 network, a final pre-scaling of approximately 0.55x is applied. For this task, too, data augmentation was applied during training. In this case, a random rotation was applied to the image, before cropping the maximum square image around the center. Other augmentation methods like arbitrary scaling have not been applied, because of absolute dimensions of the medical images.

Each network in the cross-validation was trained for 3000 epochs of 100 steps, using the Adam optimizer with a step size of 0.01 for the new layers and no adaptation for the layers taken from the Inception v3 network.

#### Evaluated approaches and configurations

In total, we evaluated three CNN-based approaches

1. **CNN/ppf@0.5x** CNN-based detection using patch probability fusion, patch size 80 × 80 px, resized image (scaling factor 0.5) (see Fig. 2)
2. **CNN/ppf@1.0x** CNN-based detection using patch probability fusion, patch size 80 × 80 px, original (unscaled) image
3. **CNN/TF@0.55x** Transfer learning approach using pre-trained CNNs, maximum square image, scaled to 224 × 224

The code for all approaches is publicly available at the following URL: http://www5.cs.fau.de/~aubreville/.

## Results

### Cross-Validation

We evaluated both the feature- and the CNN-based methods using a leave-one-patient-out cross-validation, i.e. one patient always represented the test data and all others the training data. This way, inherent correlation within the image sequences (as these were recorded as videos) did not play a role in the evaluation.

Even if the average share of carcinogen images was 53.63%, there was a huge difference in this amongst the patients ($\sigma =\mathrm{21,}\,27\, \%$). Because of this, accuracy ratings of individual patients could largely be subject to the distributions and thus be dominated by a single class. To circumvent this, all classification results from the validation are concatenated at the end of each cross-validation step, and the resulting vector is used for the comparison of the evaluated methods.

### Textural feature-based Classification

The method by Jaremenko *et al*.^19^, using textural features on image patches, yielded cross-validation accuracy ratings of 77.9% (sensitivity: 80.2%, specificity: 72.2%) on the LBP-based feature vector and 70.6% (sensitivity: 75.5%, specificity: 63.9%) on the GLCM-based feature vector, both using random forest classifiers. This performance is significantly different to the original publication. The reason for this is that the image quality in the present data set is much more mixed as more patients and more anatomical locations were considered.

**Figure 5:**
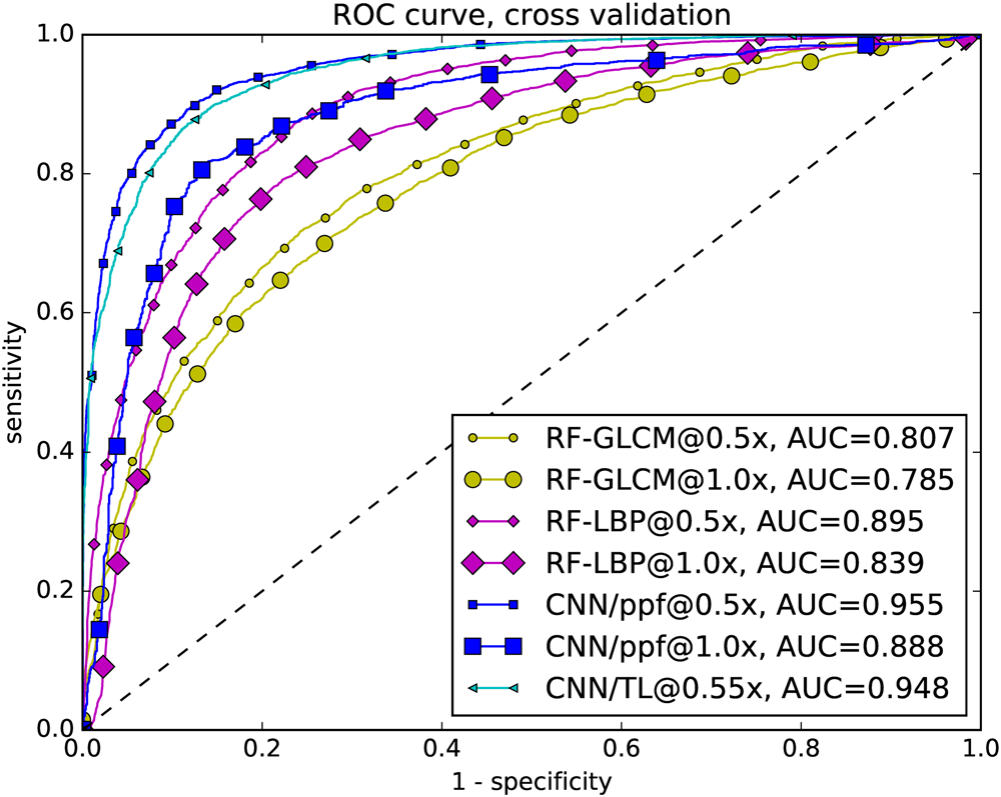
ROC curve of cross-validation. All results of the single cross-validation steps were combined into one result vector.

For both approaches, resizing the image to half the original dimensions (factor 0.5) before patch extraction yielded significant advances in detection performance: The LBP-based classifier improved to an accuracy of 81.4% (sensitivity: 84.7%, specificity: 78.2%) and the GLCM-based classifier to an accuracy of 73.1% (sensitivity: 77.5%, specificity: 69.5%). The receiver operating characteristic (ROC) curve evaluation in Fig. 5 shows the sensitivity and specificity for different discrimination thresholds. In result, the Area Under Curve (AUC) improved for the scaled patches and LBP features from 0.84 to 0.90, and for GLCM-features from 0.78 to 0.81.

### CNN-based approaches

#### Patch-probability fusion

The CNN training was performed on 40 batches, each containing around 12,000 patches. Due to the size of the data set, convergence was reached at around 60 epochs.

After fusion of the patch probabilities (as described in (Eqn. 7)), we find a leave-one-patient-out cross-validation accuracy of 88.3%, at a sensitivity of 86.6% and a specificity of 90.0%. The AUC is 0.955 (see Fig. 5).

#### Transfer Learning on the whole Image

The transfer learning approach on the maximally sized square image leads to remarkable results, given that a smaller portion of the image is considered for classification. The overall leave-one-patient out cross-validation accuracy is 87.02% at a sensitivity of 90.71% and specificity of 83.80%. As seen in Fig. 5, the area-under-curve is 0.948.

### Generalization to other anatomical locations

In order to make a first attempt into generalization of the presented ppf method, we performed an additional evaluation on a new test data set. This data set was acquired not within the oral cavity but on the vocal cords, at a different clinic within the University Hospital Erlangen and from a different medical crew. Details of these experiments are available online^40^. We found that the results on the data set reach an accuracy of 89.45% and an AUC value of 0.955, which is almost equal to approaches trained on the data set itself, which indicates a possibly strong generalization of both, the method and the data set.

## Discussion

The textural-feature based methods performed worse as expected on the data set, especially compared to findings of Vo^34^ and Jaremenko^19^. This may be explained by the much wider spread image qualities in the data set, which do however represent the clinical use case. The CNN-based method further has a greater inherent structural complexity and may be thus able to cope with different image qualities better.

Over full-image processing, the patch-extraction-based approach however reduces computational complexity significantly, as does the initial rescaling of the image. Reduced complexity will help implementation of a real-time system, and also often contributes to the robustness of a system.

**Figure 6:**
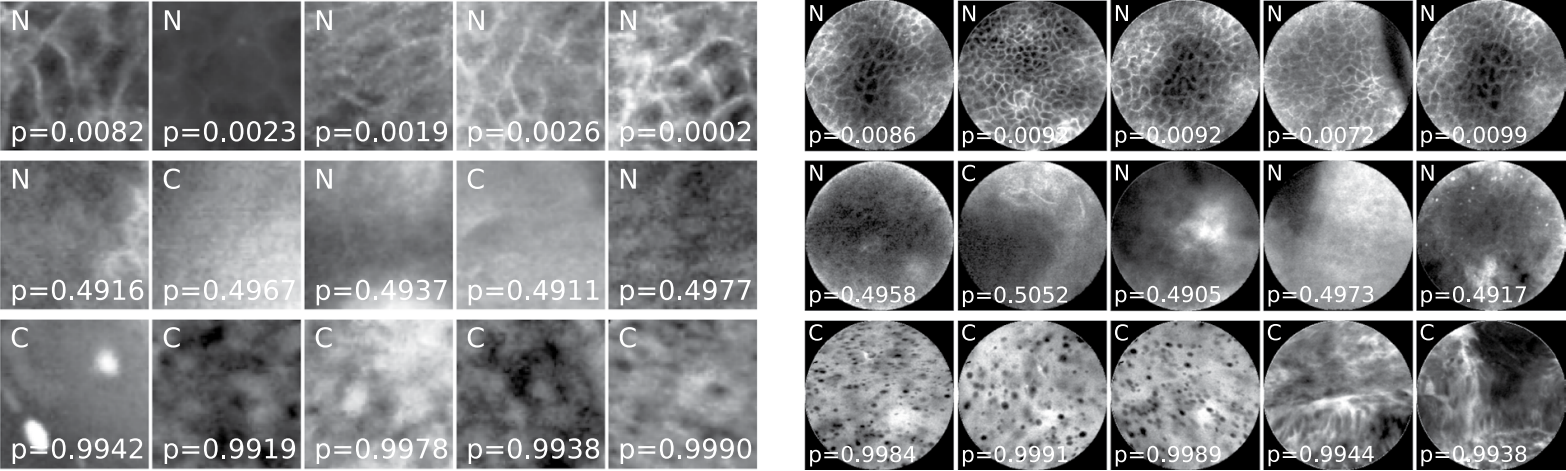
Randomly selected CNN patch **(a)** and image **(b)** classifications with overall high probability for clinically normal and carcinogenic tissue (top and bottom) and uncertain images (middle). N indicates images representing clinically normal (presumably healthy) tissue, C carcinogenic images. The probability given is the a posteriori probability for the class carcinogenic.

One common criticism about non-feature driven machine learning techniques is that the resulting networks can not be easily interpreted, and it might be unclear what weaknesses and strengths the approach has. Analysis of the patches with high probabilities for one class or the other indicate, that the convolutional neural net primarily looks for cell border structures. To illustrate this, Fig. 6 (left) shows a random pick of highly probable (as of the predicted classification probability) cancerous and clinically normal image patches, as well as images where the classifier was unsure what to choose (probability around chance). The latter would typically have rare occurrences of cell borders or no structure whatsoever. The structures assigned to being cancerous typically show signs of unorganized tissue structure like described by Oetter *et al*.^9^ or of fluorescein leakage (bright background) or cell clustering (black spots).

Analysis of images detected as being clinically normal with a high probability show typically intact cell border networks (see Fig. 6 right, top row). The images with an image class probability around chance show, why the image recognition task is sometimes even hard for experts on CLE images (Fig. 6 right, middle row). Although the images shown in the middle row of the figure are all from macroscopical normal epithelium, no clearly organized cell structures can be spotted. For the images where the classifier is sure about being cancerous (Fig. 6 right, bottom row), clear signs of carcinoma can be spotted: Fluorescein leakage as well as unorganized structure are clearly visible.

The principal drawback of rectangular patch extraction of a round image is, of course, that information at the borders is being discarded and thus not helping for the classification. We assumed that all patches of the image are highly representative of the overall image, and that the border regions can thus be neglected. However, for images where dysplastic or carcinogenic tissue characteristics are observable in these parts, inclusion of this data would be helpful.

Further, besides scaling, no preprocessing was considered at all for the images. Mualla^41^ and Bier^42^ however showed, that preprocessing can improve detection results in CLE images.

For a truly automatic assessment of CLE images, it would further be necessary to automatically annotate artifacts. Image quality-based gating should improve overall performance, as already shown for CLE images by Kamen *et al*.^17^. Especially for motion and noise artifacts, an accurate grading seems to be possible using textural descriptors. However, for anatomical structures – typically not relevant for the cancer diagnosis – this might be much more difficult and might be another interesting task for deep learning.

For the patch probability fusion method, we have chosen a rather simple network topology. However, deep learning does not stop at these basic structures but is aimed at networks with a much higher number of layers. Given enough training material, very deep approaches such as residual neural networks^43^ could certainly show beneficial behavior on the given field.

For the transfer learning approach, one main downside is certainly the limitation to a squared image in the middle of the CLE view area, which discards 36% of the available area and thus of the available information. Certainly, an approach tailored towards the round shape of CLE could lead to improvements in detection accuracy here.

One clinically very interesting task is staging of cancerous tissue - from hyperplasia over mild, moderate and severe dysplasia up to carcinoma *in situ* ^44^. The visual clinical oral examination (COE) can only be seen as a screening method for irregularities and lesions of the oral cavity^45^. Thus, the gold standard of diagnosis is the histo-pathological diagnosis of the suspicious region. Even though this method allows for a highly accurate identification of malignant oral tissue, a grading of the oral cancer as well as the identification of pre-malignant lesions and cellular dysplasia is still subject to inter-rater as well as intra-rater variabilities and thus considered as a subjective parameter with rather low reproducibility^46^.

Also, from the machine learning point of view this is a challenging task as the occurrence of intermediate stage images is usually rare and the task is much more difficult as differentiation is much harder (even for a human expert viewer). This is also due to the fact that the used CellVizio system has a fixed penetration depth, and thus tissue can only be observed in a defined 2D layer. Yet, carcinogenesis is three-dimensional, and usually originating from deeper layers and thus early stages might not be observable using the imaging system.

We focused on an accurate and reliable characterization of physiological (benign) and cancerous (malign) tissue. This work forms the basis for further studies including pre-malignant lesions as an essential step towards the identification of cancer. Other entities like precursor lesions of cancer (leukoplakia, lichen ruber etc.) or benign inflammatory reactions have to be separated towards physiological or cancerous mucosa in the future, especially with regard to the surveillance of such precursor lesions.

One principal remaining question about our data is whether the images marked as clinically normal actually all show physiological tissue. Since no histopathology was performed to assess the tissue due to ethical restrictions, there might be undiscovered pathologies in the image material. In particular, as oral cancer can be seen as a disease based on the theory of field cancerisation with occurring pre-neoplastic processes all over the oral cavity, a general alteration of the mucosa in this type of patients can not be ruled out. As all patients that were part of the study were diagnosed with HNSCC, it can thus be assumed that the prevalence of physiologically abnormal tissue is increased in this patient group. For a clinically valid procedure, it would be important to include an (age and gender matched) healthy control group, which would yet have to be recorded. Since the intravenous administration of the fluorescent agent comes with a low risk which can not be excluded fully and, above all, taking an invasive biopsy has a remaining risk of complications (such as infection, secondary bleeding or cicatrization), performing this procedure on healthy persons is ethically questionable, and it is unclear to what degree a valid and histo-pathologically correlated acquisition of this data is possible at all.

## Summary

In this work the huge potential of applying deep learning technologies to the field of cancer detection in confocal laser endomicroscopy has been outlined.

For the first time to our knowledge, image recognition based on Convolutional Neural Networks was successfully applied on CLE images of OSCC. The patch probability fusion method, described in this paper, is shown to significantly outperform the conventional approaches like image texture-based classifiers and even better than transfer learning-based image classification using CNNs.

This work represents a significant step towards an automatic identification of cancerous lesions in CLE imaging, for which generalization on more patients and other tissue have yet to be shown. Such an automatic real-time tool, however, could improve the conventional clinical workflow of visual and tactile screening for oral cancer. Moreover, an accurate diagnostic test directly on site would accurately identify and outline high-risk regions that need further investigations by the current gold standard of an invasive biopsy and histopathological assessment.

This study shows a great prospect not only for the CLE imaging of carcinomas in the oral cavity, as squamous cells are omnipresent in the mucosa of the upper aero-digestive and respiratory tract. Further studies have to be conducted to a) expand the present findings to the more complex task of identification and differentiation of pre-malignant lesions *in situ* and to b) transfer the findings to further entities of squamous cell carcinomas in the upper aero-digestive tract. Additionally, the task of a real-time identification of OSCC directly on the patient during the screening process will be pursued by our workgroup by optimization of the underlying mathematical algorithms.

## References

[1] Forastiere A, Koch W, Trotti A, Sidransky D (2001). Head and Neck Cancer. The New England Journal of Medicine.

[2] Ferlay J (2014). Cancer incidence and mortality worldwide: Sources, methods and major patterns in GLOBOCAN 2012. International Journal of Cancer.

[3] Muto M (2004). Squamous cell carcinoma *in situ* at oropharyngeal and hypopharyngeal mucosal sites. Cancer.

[4] Swinson B, Jerjes W, El-Maaytah M, Norris P, Hopper C (2006). Optical techniques in diagnosis of head and neck malignancy. Oral oncology.

[5] Knipfer C (2014). Raman difference spectroscopy: a non-invasive method for identification of oral squamous cell carcinoma. Biomedical Optics Express.

[6] Laemmel E (2004). Fibered confocal fluorescence microscopy (Cell-viZio) facilitates extended imaging in the field of microcirculation. A comparison with intravital microscopy. Journal of vascular research.

[7] Neumann H, Kiesslich R, Wallace MB, Neurath MF (2010). Confocal Laser Endomicroscopy: Technical Advances and Clinical Applications. YGAST.

[8] Hoffman A (2006). Confocal laser endomicroscopy: technical status and current indications. Endoscopy.

[9] Oetter N (2016). Development and validation of a classification and scoring system for the diagnosis of oral squamous cell carcinomas through confocal laser endomicroscopy. Journal of Translational Medicine.

[10] Nathan CAO (2014). Confocal Laser Endomicroscopy in the Detection of Head and Neck Precancerous Lesions. Otolaryngology – Head and Neck Surgery.

[11] Helmchen F (2002). Miniaturization of fluorescence microscopes using fibre optics. Experimental Physiology.

[12] Minsky M (1988). Memoir on inventing the confocal scanning microscope. Scanning.

[13] Abbaci, M., Breuskin, I., Casiraghi, O. & De Leeuw, F. Confocal laser endomicroscopy for non-invasive head and neck cancer imaging: a comprehensive review. *Oral oncology* (2014). 10.1016/j.oraloncology.2014.05.002 24932530

[14] Neumann H, Vieth M, Atreya R, Neurath MF, Mudter J (2011). Prospective evaluation of the learning curve of confocal laser endomicroscopy in patients with IBD. Histology and histopathology.

[15] Mennone A, Nathanson M (2011). Needle-based confocal laser endomicroscopy to assess liver histology *in vivo*. Gastrointestinal Endoscopy.

[16] André B (2012). Software for automated classification of probe-based confocal laser endomicroscopy videos of colorectal polyps. World journal of Gastroenterology.

[17] Kamen A (2016). Automatic Tissue Differentiation Based on Confocal Endomicroscopic Images for Intraoperative Guidance in Neurosurgery. BioMed Research International.

[18] Veronese, E. *et al*. Hybrid patch-based and image-wide classification of confocal laser endomicroscopy images in Barrett’s esophagus surveillance. In *2013 IEEE 10th International Symposium on Biomedical Imaging (ISBI 2013)*, 362–365 (IEEE, 2013).

[19] Jaremenko, C. *et al*. Classification of Confocal Laser Endomicroscopic Images of the Oral Cavity to Distinguish Pathological from Healthy Tissue. In *Bildverarbeitung für die Medizin 2015*, 479–485 (Springer Berlin Heidelberg, 2015).

[20] Dittberner A (2016). Automated analysis of confocal laser endomicroscopy images to detect head and neck cancer. Head & Neck.

[21] Rodner, E. *et al*. Analysis and Classification of Microscopy Images with Cell Border Distance Statistics. In *Jahrestagung der Deutschen Gesellschaft für Medizinische Physik DGMP*, 1–2 (2015).

[22] Hubel DH, Wiesel TN (1968). Receptive fields and functional architecture of monkey striate cortex. The Journal of physiology.

[23] Russakovsky O, Deng J, Su H, Krause J (2015). Imagenet large scale visual recognition challenge. International Journal of Computer Vision.

[24] Shin H-C (2016). Deep Convolutional Neural Networks for Computer-Aided Detection: CNN Architectures, Dataset Characteristics and Transfer Learning. IEEE Transactions on Medical Imaging.

[25] Roth HR (2016). Improving Computer-Aided Detection Using Convolutional Neural Networks and Random View Aggregation. IEEE Transactions on Medical Imaging.

[26] Esteva A (2017). Dermatologist-level classification of skin cancer with deep neural networks. Nature.

[27] Litjens G (2016). Deep learning as a tool for increased accuracy and efficiency of histopathological diagnosis. Nature Publishing Group.

[28] Würfl, T., Ghesu, F. C., Christlein, V. & Maier, A. Deep Learning Computed Tomography. In *Medical Image Computing and Computer-Assisted Intervention – MICCAI 2016*, 432–440 (Springer International Publishing, Cham, 2016).

[29] Aubreville, M. *et al*. Correlation-based Alignment of Raw Endoscopic Sequence Data with Physician Selected Movies. *Workshop Germany Brazil 2016: Understanding the aggressiveness of cancer cells through novel imaging techniques* (2016).

[30] Ahonen T, Hadid A, Pietikäinen M (2006). Face Description with Local Binary Patterns: Application to Face Recognition. IEEE transactions on pattern analysis and machine intelligence.

[31] Nanni L, Lumini A, Brahnam S (2010). Local binary patterns variants as texture descriptors for medical image analysis. Artificial intelligence in medicine.

[32] Haralick RM, Shanmugam K, Dinstein I (1973). Textural Features for Image Classification. IEEE Transactions on Systems, Man, and Cybernetics.

[33] Baraldi A, Parmiggiani F (1995). An investigation of the textural characteristics associated with gray level cooccurrence matrix statistical parameters. IEEE Transactions on Geoscience and Remote Sensing.

[34] Vo, K., Jaremenko, C., Maier, A., Neumann, H. & Bohr, C. Automatic Classification and Pathological Staging of Confocal Laser Endomicroscopic Images of the Vocal Cords. *Bildverarbeitung für die Medizin 2017*, 312 (Springer Berlin Heidelberg, 2017).

[35] Lecun Y, Bottou L, Bengio Y, Haffner P (1998). Gradient-based learning applied to document recognition. Proceedings of the IEEE.

[36] Abadi, M. *et al*. TensorFlow: A system for large-scale machine learning. *ArXiv e-prints* (2016).

[37] Kingma, D. & Ba, J. Adam: A method for stochastic optimization. *ICLR 2015, reprint on arXiv.org* (2014).

[38] Szegedy, C., Vanhoucke, V., Ioffe, S., Shlens, J. & Wojna, Z. Rethinking the Inception Architecture for Computer Vision. In *2016 IEEE Conference on Computer Vision and Pattern Recognition (CVPR)*, 2818–2826 (IEEE, 2016).

[39] Deng, J. *et al*. ImageNet: A Large-Scale Hierarchical Image Database. In *2009 IEEE Conference on Computer Vision and Pattern Recognition (CVPR)* (2009).

[40] Aubreville, M. *et al*. Patch-based Carcinoma Detection on Confocal Laser Endomicroscopy Images - A Cross-Site Robustness Assessment. *ArXiv e-prints* (2017).

[41] Mualla, F., Schöll, S., Bohr, C., Neumann, H. & Maier, A. Epithelial Cell Detection in Endomicroscopy Images of the Vocal Folds. In *International Multidisciplinary Microscopy Congress*, 201–205 (Springer International Publishing, Cham, 2014).

[42] Bier, B. *et al*. Band-Pass Filter Design by Segmentation in Frequency Domain for Detection of Epithelial Cells in Endomicroscope Images. In *Bildverarbeitung für die Medizin 2015*, 413–418 (Springer Berlin Heidelberg, 2015).

[43] He, K., Zhang, X., Ren, S. & Sun, J. Deep Residual Learning for Image Recognition. In *2016 IEEE Conference on Computer Vision and Pattern Recognition (CVPR)*, 770–778 (IEEE, 2016).

[44] Keith RL, Miller YE (2013). Lung cancer chemoprevention: current status and future prospects. Nature Reviews Clinical Oncology.

[45] Cleveland JL, Robison VA (2013). Clinical oral examinations may not be predictive of dysplasia or oral squamous cell carcinoma. The journal of evidence-based dental practice.

[46] Abbey LM, Kaugars GE, Gunsolley JC, Burns JC (1995). Intraexaminer and interexaminer reliability in the diagnosis of oral epithelial dysplasia. Oral Surgery.

